# Valproic acid overcomes hypoxia-induced resistance to apoptosis

**DOI:** 10.3892/or.2011.1577

**Published:** 2011-12-06

**Authors:** ŠIMON CIPRO, JANA HŘEBAČKOVÁ, JAN HRABĚTA, JITKA POLJAKOVÁ, TOMÁŠ ECKSCHLAGER

**Affiliations:** 1 Department of Pediatric Hematology and Oncology, 2nd Medical School, Charles University and University Hospital Motol, V Úvalu 84, 150 00 Prague 5; 2 Department of Biochemistry, Faculty of Science, Charles University, Albertov 2030, 128 40 Prague 2, Czech Republic

**Keywords:** valproic acid, hypoxia, apoptosis, neuroblastoma

## Abstract

Valproic acid (VPA), a histone deacetylase inhibitor (HDACi), has been shown to be an effective tool in cancer treatment. Although its ability to induce apoptosis has been described in many cancer types, the data come from experiments performed in normoxic (21% O_2_) conditions only. Therefore, we questioned whether VPA would be equally effective under hypoxic conditions (1% O_2_), which is known to induce resistance to apoptosis. Four neuroblastoma cell lines were used: UKF-NB-3, SK-N-AS, plus one cisplatin-resistant subline derived from each of the two original sensitive lines. All were treated with VPA and incubated under hypoxic conditions. Measurement of apoptosis and viability using TUNEL assay and Annexin V/propidium iodide labeling revealed that VPA was even more effective under hypoxic conditions. We show here that hypoxia-induced resistance to chemotherapeutic agents such as cisplatin could be overcome using VPA. We also demonstrated that apoptosis pathways induced by VPA do not differ between normoxic and hypoxic conditions. VPA-induced apoptosis proceeds through the mitochondrial pathway, not the extrinsic pathway (under both normoxia and hypoxia), since inhibition of caspase-8 failed to decrease apoptosis or influence bid cleavage. Our data demonstrated that VPA is more efficient in triggering apoptosis under hypoxic conditions and overcomes hypoxia-induced resistance to cisplatin. The results provide additional evidence for the use of VPA in neuroblastoma (NBL) treatment.

## Introduction

Neuroblastoma (NBL) is the most common extracranial solid tumor in children and a major cause of neoplastic death in infancy. It originates from undifferentiated cells of the sympathetic nervous system. Based on its cellular and biological heterogeneity, NBL behavior can range from low-risk cancers with a tendency toward spontaneous regression or maturation, to high-risk cancers with extensive growth, early metastasis and a poor prognosis (1). Treatment of high-risk neuroblastomas (HR NBL) usually fails despite intensive therapy, which includes megatherapy followed by hematopoietic progenitor cell transplantation, biotherapy and immunotherapy. Treatment failure is due to drug resistance that arises in the majority of patients who initially responded well to chemotherapy. The necessity to develop new treatment modalities is indisputable.

An increasing body of information indicates that epigenetic modifications are associated with cancer onset and progression. This awareness has led to prolific research into drugs that interfere with the epigenome (2,3). Histone deacetylase inhibitors (HDACi) represent such a group of compounds since histones are the main protein components of chromatin and have an indispensable role in gene regulation. Cancer cell histones are frequently hypo-acetylated, due to overexpression of histone deacetylases (HDACs), and are often connected with impaired gene transcription in tumors (4), including dysregulation of genes responsible for growth control and apoptosis. Consequently inhibition of HDACs can reactivate gene transcription and restore the balance between pro- and anti-apoptotic genes and eventually lead to apoptosis (5). HDAC inhibition also decompacts chromatin structure making the DNA structure more available to other cytotoxic agents that target DNA. Despite advances in understanding, the mode of anti-tumor action of HDACi is complex and still not completely understood (6,7).

Valproic acid (VPA) has been studied as an anti-cancer drug excessively over the past years because it can be taken orally, is well tolerated by patients and there is cumulative experience coming from its use as an anti-epileptic drug. Although earlier reports showed the cytotoxic potential of VPA on NBL cells *in vitro* and *in vivo* (8,9), the studies were carried out solely under normoxic conditions and little was known about its anti-tumor activity under hypoxic conditions.

Hypoxic areas are common in solid tumors. Hypoxia arises as a consequence of pathological microcirculation within the tumor. Rapid tumor growth can outstrip its own blood supply and therefore cancer cells are exposed to oxygen deprivation (chronic hypoxia) (10). Another factor that contributes to tumor hypoxia is the poor quality of the newly developing tumor vessels, which often display severe structural abnormalities. Whereas normal vasculature shows a hierarchical branching pattern, tumor blood vessels are often tortuous in appearance with uneven diameters, branch irregularity and form arterio-venous shunts. These vessels are more susceptible to thrombosis and on occasion collapse, which ultimately leads to acute hypoxia within the tumor mass (11).

Hypoxia also induces adaptational changes in cells that are otherwise physiological, in the sense that they are normal and noncancerous; however, due to regional hypoxia these cells contribute to chemo- and radio-resistance in hypoxic cancer cells (12–14). Notably, hypoxia-induced resistance is not limited to only conventional chemotherapy but it can also decrease the efficiency of targeted therapy, as documented with imatinib in cases of chronic myeloid leukemia (15). Additionally, hypoxia induces genomic instability that leads to progressive transformation of cancer cells into more malignant phenotypes (16). The presence of hypoxic regions within the tumor mass correlates with more aggressive phenotypes, lower response rates and a decline in overall disease survival (17–19).

In our study, we addressed the issue of whether hypoxia promotes resistance to VPA and if apoptosis pathways differ between normoxic and hypoxic conditions, with respect to VPA treatment.

## Materials and methods

### Cell lines and chemicals

The UKF-NB-3 cell line was established from bone marrow metastases of HR NBL with *MYCN* amplification. The line was kindly provided by Professor J. Cinatl Jr. (Institute for Medical Virology, Hospital of the Johann Wolfgang Goethe University, Frankfurt, Germany). Cells were grown in Iscove’s modified Dulbecco’s medium (IMDM) with 10% fetal calf serum (PAA Laboratories, Pasching, Austria). The SK-N-AS cell line was derived from bone marrow metastasis of a female patient with HR NBL. SK-N-AS, with normal diploid *MYCN* status, was purchased from the European Collection of Cell Cultures (ECACC, Salisbury, UK) and was cultivated according to the manufacturer’s instructions. The CDDP-resistant sub-line, designated UKF-NB-3^CDDP^ was also kindly provided by Professor J. Cinatl Jr. SK-N-AS^CDDP^ was prepared in our laboratory by incubation of parental cells with increasing concentrations of CDDP. Solutions of CDDP (EBEWE Pharma Ges.m.b.H. Nfg. KG, Unterach, Austria) were prepared according to the manufacturer’s instructions. CDDP-resistant cell lines were cultivated in a medium containing 1 μg/ml of CDDP. Valproic acid (dissolved in distilled water) and trichostatin A (dissolved in DMSO) were purchased from Sigma Chemical Co. (St. Louis, MO, USA). The specific caspase-8 inhibitor, Z-IETD-FMK (specific caspase-8 inhibitor), was obtained from R&D Systems, Inc. (Minneapolis, MN, USA). It was dissolved in DMSO and was used at a final concentration of 2 μM, as recommended by producer. All other chemicals used in experiments were of analytical purity or better.

### Hypoxic environment

A hypoxia chamber purchased from Billups-Rothenberg (Del Mar, CA, USA) was prepared with an atmosphere containing 1% O_2_, 5% CO_2_, and 94% N_2_. Controls were grown at 5% CO_2_ and all samples were grown at 37°C.

### Annexin V/propidium iodide labeling

Annexin V, a phospholipid-binding protein with a high affinity for phosphatidyl serine, was used to measure apoptosis and viability. Apoptosis was determined using an Annexin V-FITC Apoptosis Detection kit according to manufacturer instructions (Biovision, Mountain View, CA, USA). Cells were washed in PBS and resuspended in a ‘binding buffer’ after incubation with different compounds, under normoxic and/or hypoxic conditions, as described below. Cells were incubated with Annexin V and propidium iodide for 10 min at room temperature and then analyzed using flow cytometry (FACSCalibur, BD, San Jose, CA, USA). Data obtained from flow cytometry were evaluated using the same technique described in a study by Bossy-Wetzel (20).

### TUNEL assay

Apoptotic cells were determined using an ApoDirect DNA Fragmentation Assay kit per manufacturer’s instructions (Biovision). Cells were fixed with 1% paraformaldehyde and then incubated with terminal deoxynucleotidyl transferase and FITC-dUTP for 60 min at 37°C and counter-stained with propidium iodide. Cells were then analyzed using flow cytometry.

### Western blot was used to determine the expression of BID protein

Cells were homogenized in RIPA buffer. Protein concentrations were assessed using the DC protein assay (Bio-Rad, Hercules, CA, USA) with serum albumin as a standard. 10–45 μg of extracted proteins were subjected to SDS-PAGE electrophoresis on a 10% gel. After migration, proteins were transferred to a nitrocellulose membrane and incubated with 5% non-fat milk to block non-specific binding. The membranes were then exposed to specific anti-BID (1:1000, AbCam, Cambridge, UK ) rabbit monoclonal antibodies overnight at 4°C. Membranes were washed and exposed to peroxidase-conjugated anti-IgG secondary antibody (1:3000, Bio-Rad), and the antigen-antibody complex was visualized using an enhanced chemiluminescence detection system according to the manufacturer’s instructions (Immun-Star HRP Substrate, Bio-Rad). The resulting films (MEDIX XBU, Foma, Hradec Králové, Czech Republic) were scanned with a computerized image-analyzing system (ElfoMan 2.0, Ing. Semecký, Prague, Czech Republic).

### Caspase activity

Caspase-8 activity was measured using a caspases-8 assay kit according to manufacturer’s instructions (Biovision). Briefly, cells were lysed in cell lysis buffer after incubation with VPA. Total protein (200 μg) were added to the reaction buffer, which contained IETD-pNA colorimetric substrate, and incubated for 2 h at 37°C. Hydrolyzed pNA was detected using a VersaMax plate reader (Molecular Device Inc., Sunnyvale, CA, USA) at 405 nm.

### Real-time PCR analysis

Total RNA was extracted from cells lines using TRIzol reagent (Invitrogen, Carlsbad, CA, USA). The quality of the isolated RNA was verified using horizontal agarose gel electrophoresis and RNA quantity was measured using a BioMate 3 UV-Vis Spectrophotometer (Thermo Scientific, Waltham, MA, USA). Complementary DNA was synthesized from 500 ng of RNA using random hexamers and MultiScribe reverse transcriptase (Applied Biosystems, Foster City, CA, USA). RT-PCR was performed using assays for vascular endothelial growth factor (VEGF), carbonic anhydrase-9 (CA9) and β-2-microglobulin (B2M) purchased from Generi Biotech (Hradec Kralove, Czech Republic). B2M was used as a reference gene. Relative expression and statistical significance were determined using REST-MCS software (Dr Michael Pfaffl, Germany) using the technique described by Pfaffl (21).

## Results

### VPA induces apoptosis under both normoxic and hypoxic conditions

**Figure 1:**
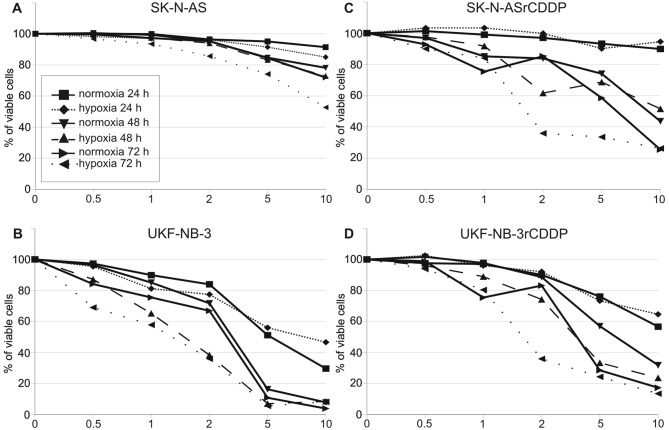
Cell viability measured as An^−^/PI^−^ cells. Maternal cell lines SK-N-AS and UKF-NB-3 (A and B) and cell lines resistant to cisplatin (rCDDP) derived from them (C and D). Cells were grown under normoxic conditions for 24 h before administration of VPA.

We set up dose and time course experiments in order to prove efficacy of VPA under hypoxic and normoxic conditions. Concentrations of VPA ranged from 0.5 to 10 mM. Cells were grown under normoxic conditions for 24 h after plating and then VPA was added. Plates were then put into the hypoxia chamber, while control cells stayed under normoxic conditions. Apoptosis was determined using Annexin V (An) and propidium iodide (PI) staining at 24, 48 and 72 h after addition of VPA. We observed time- and dose-dependent apoptosis. UKF-NB-3 showed higher sensitivity to VPA compared to SK-N-AS (Fig. 1A and B). We did not observe any hypoxia induced resistance to VPA. Moreover, slightly more Annexin positive/propidium iodide negative cells (early apoptotic) and Annexin positive/propidium iodide positive cells (late apoptotic or necrotic) were seen under hypoxic conditions in both cell lines (Table I). For instance, 13.4% Annexin V single positive (An^+^/PI^−^) cells were observed after treatment with 5 mM VPA under normoxic conditions whereas 19.0% An^+^/PI^−^ cells were observed in the hypoxia SK-N-AS cell line. Although the higher number of apoptotic cells, under hypoxic conditions, was not statistically significant, this trend was clearly obvious in all cell lines tested. This result indicates that VPA promotes apoptosis irrespective of oxygen tension and therefore should be equally efficient throughout the entire tumor volume. We performed the same experiments with cell lines resistant to cisplatin, which had been derived from SK-N-AS and UKF-NB-3, and obtained similar results (Fig. 1C and D).

We also evaluated apoptosis using TUNEL assay in order to validate the data using an independent method. Both SK-N-AS and UKF-NB-3 cell lines revealed higher number of apoptotic cells (TUNEL positive) under hypoxic conditions than under normoxic conditions. The TUNEL results therefore supported the data obtained using An/PI staining (data not shown).

### VPA has a synergistic effect with cisplatin

As mentioned in a previous section, VPA is capable of overcoming hypoxia resistance; however, its overall toxicity to NBL cells is quite poor considering that clinically achievable concentrations are <1 mM. Thus, we addressed the issue of whether small concentrations of VPA, which are clinically well tolerated, could be useful in overcoming hypoxia induced resistance to chemotherapeutic agents, such as cisplatin (CDDP), which are commonly used in HR NBL therapy.

**Figure 2:**
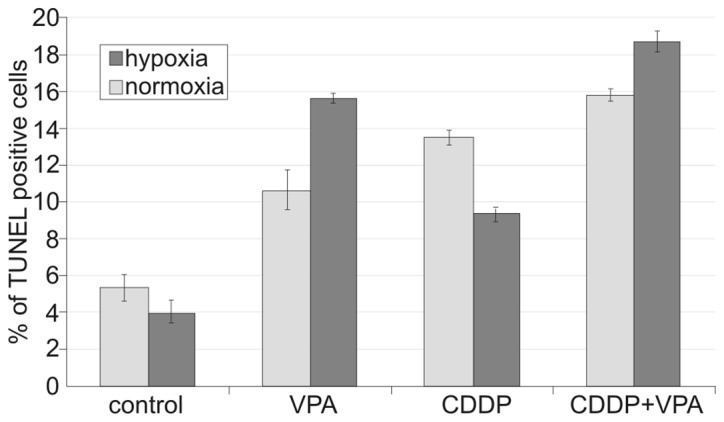
VPA synergizes with cisplatin (CDDP) under hypoxic conditions. UKF-NB-3 cells were exposed to 1 mM VPA and 1 μM CDDP at the same time. One representative experiment is shown.

Cells were treated with lower concentrations of VPA (1 mM) or CDDP (1 μM) alone and in combination. Apoptosis was assessed 24 h after administration of the drugs using a TUNEL assay. The degree of apoptosis induced by CDDP alone was diminished by hypoxic conditions, while VPA alone was more efficient under hypoxic conditions than under normoxic conditions. Cells administered as combination of VPA and CDDP showed a higher degree of apoptosis under hypoxic conditions (Fig. 2), suggesting not merely a synergistic effect for VPA and CDDP, but the added ability of VPA to overcome hypoxia-induced resistance to CDDP.

### VPA activates caspase-8

**Figure 3:**
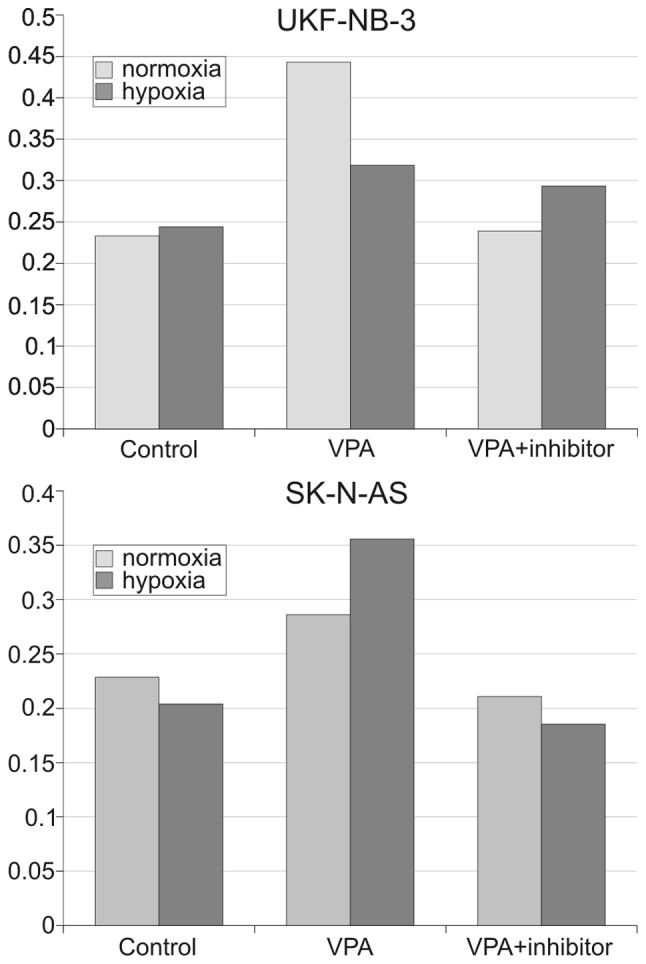
Caspase-8 activity and VPA treatment. VPA increased activity of caspase-8 in both parental cell lines (UKF-NB-3 and SK-N-AS).

To clarify whether VPA activates the receptor-mediated apoptotic pathway, we determined the activity of caspase-8. Cells were grown for 24 h and then 2 mM VPA was added to UKF-NB-3 cells and 5 mM was added to SK-N-AS cells. Caspase-8 activity was determined after 48 h of treatment. VPA increased the activity of caspase-8 in both cells lines (Fig. 3). Of note, caspase-8 activity was higher under hypoxic conditions in the SK-N-AS line, albeit only slightly. This discovery supports the above mentioned observations that showed VPA to be more effective under hypoxic conditions. This result also suggests that caspase-8 is the first caspase activated in the apoptotic cascade during VPA treatment, which is why we focused on the cleavage of the pro-apoptotic BID protein. Since BID is the substrate for caspase-8, its cleavage would clearly demonstrate the presence of activated caspase-8.

### VPA initiates cleavage of BID

**Figure 4:**
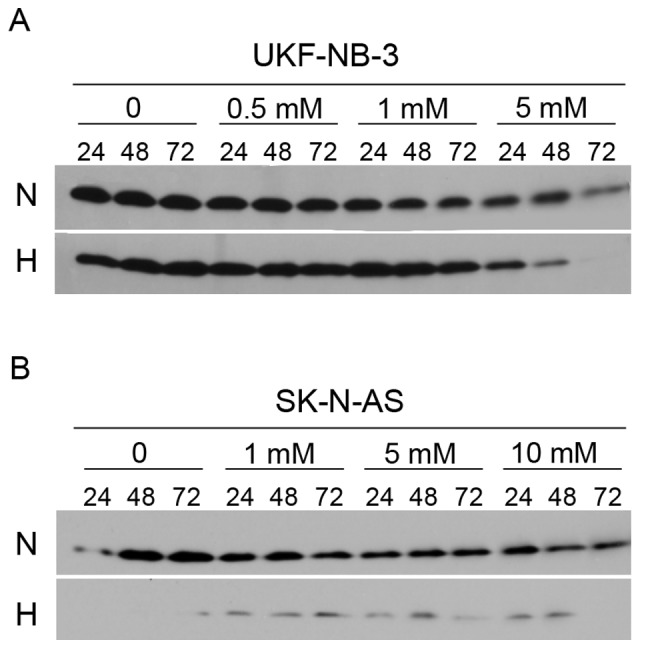
(A) Cells were incubated with different concentrations of VPA (0.5, 1 and 5 mM) for 24–72 h, this led to a decrease of full-length BID in a dose- and time-dependent manner in UKF-NB-3 under normoxic conditions (N), whereas it was cleaved only upon treatment with high concentration of VPA under hypoxic conditions (H). (B) Cleavage of bid was less expressed under normoxic conditions (N) in SK-N-AS. There was almost no detectable amount of bid under hypoxic conditions (H) in SK-N-AS.

We addressed the question whether BID is cleaved to its active form, which could consecutively activate the mitochondrial apoptotic pathway. Cells were treated with different concentrations of VPA (0.5, 1 and 5 mM for UKF-NB-3 and 1, 5 and 10 mM for SK-N-AS) for 24, 48 and 72 h (Fig. 4A). We observed a time- and dose-dependent cleavage of BID in the UKF-NB-3 cell line under normoxic conditions. Whereas under hypoxic conditions BID was cleaved only when treatment with a relatively high concentration of VPA (5 mM). In the case of the SK-N-AS line, corresponding concentrations of VPA also led to a decrease of full-length BID albeit only marginally (Fig. 4B). This is in concert with the lower overall sensitivity of this cell line to VPA. We used 20 mM of VPA to confirm the dose-dependent manner of BID cleavage in SK-N-AS. This enormous concentration of VPA, exceeding IC_50_ values of SK-N-AS, decreased full-length BID, confirmed that BID cleavage caused by VPA was really dose-dependent and also demonstrated the poor sensitivity of this cell line to VPA. Together these data indicate that BID is cleaved upon VPA treatment and can subsequently transfer the apoptotic signal from the receptor-mediated to the intrinsic apoptotic pathway.

### Inhibition of caspase-8 does not influence VPA-induced apoptosis

**Figure 5:**
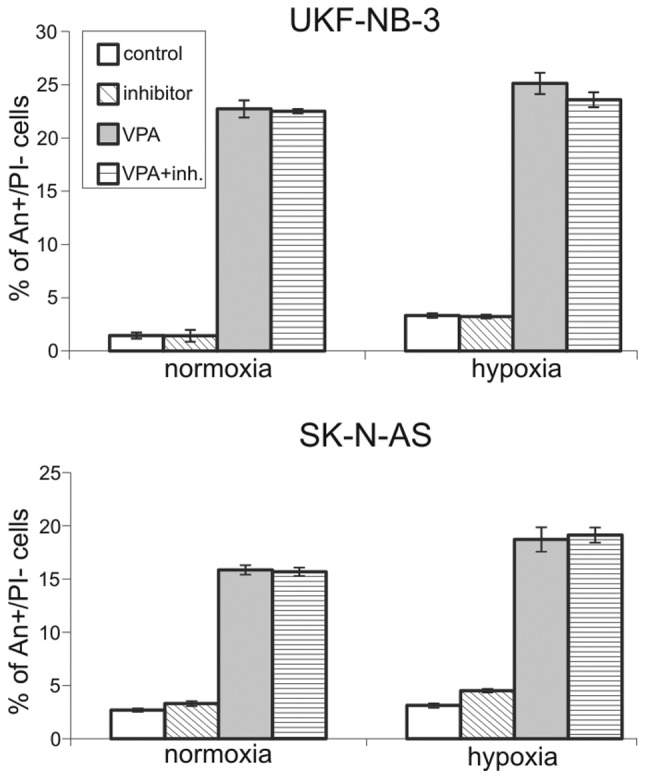
Inhibition of caspase-8 did not influence apoptosis in UKF-NB-3 or in SK-N-AS. Cells were preincubated with 2 μM of caspase-8 inhibitor for 15 min before VPA was added. Graphs shows number of apoptotic cells measured as An^+^/PI^−^ cells.

**Figure 6:**
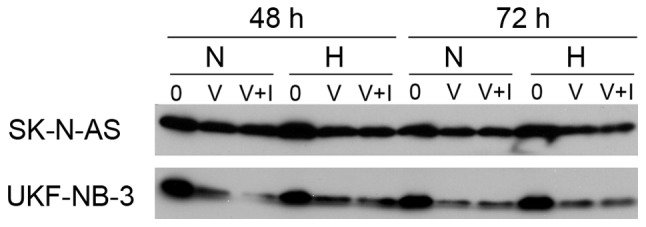
Cleavage of bid upon treatment with VPA (V) was not influenced by caspase-8 inhibitor (I). VPA (5 mM) was used for UKF-NB-3 and 10 mM for SK-N-AS.

Caspases-8 has been reported as the main effector responsible for BID cleavage (22). We therefore inhibited caspase-8 using a specific inhibitor, z-IETD-fmk, to determine whether its inhibition was capable of blocking apoptosis induced by VPA. Cells were treated with 2 μM z-IETD-fmk for 15 min preceding VPA addition. Cell cultures were then incubated together with caspase-8 inhibitor and VPA for 48 h. We employed Annexin V/PI labeling to detect apoptotic changes. Surprisingly, overall viability measured as Annexin V/PI double negative cells was not increased in samples treated with the caspase-8 inhibitor. This inhibition did not influence the percentage of early apoptotic cells (An^+^/PI^−^) (Fig. 5) nor the percentage of necrotic/late apoptotic cells (An^+^/PI^+^). We did not observe a shift of Annexin V/propidium iodide double positive cells to the Annexin V single positive population, which would have signaled that caspase-8 inhibition only delayed apoptotic progress. Moreover, there were no differences between normoxic and hypoxic conditions. WB analysis showed that BID was cleaved regardless of caspase-8 inhibition (Fig. 6), which further points to a non-essential role for caspase-8 in apoptosis induction.

The effectivity of caspase-8 inhibition was also determined by measuring its activity after treatment with z-IETD-fmk. It was found that it was decreased to the level of untreated samples (data not shown); this confirmed that the concentration of z-IEDT-fmk used was sufficient. It is therefore evident that inhibition of caspase-8 has no significant effect on apoptosis and BID cleavage in NBL cell lines.

### VPA decreases transcriptional activity of HIF-1

**Figure 7:**
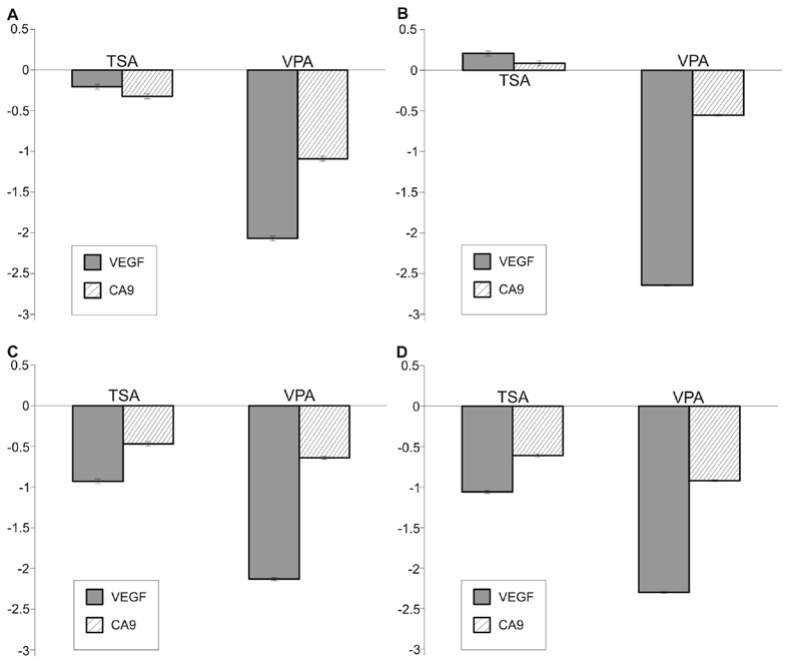
VPA and TSA decreased expression of HIF-1 target genes in both UKF-NB-3 (A and B) and SK-N-AS (C and D) after being cultivated for 3 h (A and C) and 8 h, respectively (B and D) under hypoxic conditions.

Hypoxia inducible factor 1 (HIF-1) influences the expression of many genes which can directly or indirectly inhibit apoptosis (23,24). HDACi have been described to attenuate stability of HIF-1 hence re-establishing sensitivity to apoptosis. We employed real-time PCR techniques for determination of mRNA levels of two well-described (25,26) HIF-1 target genes, VEGF and carbonic-anhydrase 9 (CA9) in order to assess whether VPA diminish HIF-1 transcriptional activity in NBL cells. Cells were preincubated with 2 mM VPA or 100 μM trichostatin A for 24 h and then placed into a hypoxia chamber for 3 and 8 h, respectively. Expression of both genes was significantly (P<0.01) decreased, in a time-dependent manner in both SK-N-AS and UKF-NB-3 cell lines (Fig. 7). VPA attenuated expression of VEGF 2.2-fold and CA9 4.2-fold compared with untreated samples of UKF-NB-3 after 3 h of hypoxia. Similar results were obtained for SK-N-AS cells. These results indicate that inhibition of HIF-1 by VPA participates with higher efficiency of VPA under hypoxic conditions by sensitizing NBL cells to apoptosis as discussed below.

## Discussion

Hypoxia is regarded as a negative prognostic factor for solid tumors. It correlates with higher risk of cancer malignancy, resistance to radio- and chemotherapy and poorer patient outcomes (27,28). Hence, agents capable of overcoming hypoxia resistance would be beneficial for cancer treatment. We found that VPA was able to induce apoptosis under hypoxic conditions and moreover, was even more efficient than under normoxic conditions. To our knowledge this is the first observation of increased VPA efficacy under hypoxic conditions. Moderate hypoxia (1% O_2_) caused apoptosis resistance in hypoxic cells (29,30). Resistance can be caused by both HIF-1-dependent and -independent mechanisms. The role of HIF-1 as an anti- or pro-apoptotic transcription factor is still controversial (31). It is dependent on the severity and duration of hypoxia, HIF-1 phosphorylation status and cell type (32). HDACi have been previously reported to attenuate HIF-1 transcription activity (33). In concert with this observation, we showed that two HDACi (VPA and TSA) down-regulate expression of HIF-1 target genes VEGF and CA9 in hypoxic NBL cells. Several mechanisms can be proposed by which inhibition of HIF-1 by VPA promotes apoptosis under hypoxic conditions via attenuation of HIF-1 transcriptional activity.

p53 is usually said to be stabilized by HIF-1 (34) hence promoting apoptosis. However, it has been recently shown that HIF-1 can also antagonize p53 pro-apoptotic function through several mechanisms. First, HIF-1 increases expression of tyrosinase-related protein 2 (TRP2; also called DCT) which then down-regulates p53, thereby impeding apoptosis (35). Second, homeodomain-interacting protein kinase-2 (HIPK2) is an important co-activator of p53. HIF-1 increases proteasomal degradation of HIPK2 under hypoxic conditions, which eventually attenuates p53 pro-apoptotic function (36). Taken together, inhibition of HIF-1 by VPA can promote apoptosis by both re-establishing HIPK2 levels and attenuation of TRP2 expression.

AP-1 is another transcription factor induced by hypoxia. Recent studies showed that induction of AP-1 is also involved in hypoxia induced resistance to apoptosis (37–39). On the other hand, we do not suspect a role for AP-1 regarding the higher efficacy of VPA during hypoxic conditions, since it has been shown that VPA enhances AP-1 mediated gene expression in the SH-SY5Y NBL cell line (40). Therefore, VPA acts, most likely, as an inductor of AP-1 rather than a suppressor. Additionally, lithium chloride (LiCl) also increases transcription activity of AP-1, however, we did not observe higher efficacy of LiCl during hypoxia (data not shown). It is therefore probable that AP-1 has no significant role in VPA induced apoptosis during hypoxia. The actual contribution of different transcription factors to hypoxia-induced apoptosis resistance depends on several things (e.g. cell type, severity and length of hypoxia and/or type of pro-apoptotic stimuli); therefore a substantial role for HIF-1 is very likely in NBL cell lines.

Two points concerning the question of whether VPA should be used as monotherapy or in a combination regimen need to be addressed. First, despite the ability of VPA to overwhelm hypoxia resistance, sensitivity of some NBL cell lines, e.g. SK-N-AS in this study and UKF-NB-4, reported in our previous study (41) is quite low. For example, there was only a 20% induction of apoptotic cells by 5 mM VPA in SK-N-AS after 72 h, whereas 1 mM VPA has been reported to induce apoptosis in >50% of cells in some hematological malignancies (5). Second, plasma levels of VPA, in patients treated for epilepsy, usually do not exceed 0.7 mM and have minimal or no side effects in such concentrations. Serious adverse reactions are seen when the concentration exceeds 3.1 mM (42). It can be argued that unlike epilectic patients, where very long-term therapy is necessary, cancer patients could tolerate short-term application of higher doses of VPA. Our measurement of apoptosis when cells were treated with VPA and CDDP together demonstrated that low concentration of VPA (1 mM) were enough to overcome hypoxia induced apoptosis resistance to CDDP while still maintaining low VPA toxicity. Based on this we see VPA, in NBL treatment, mainly used in combination regimens in which its low concentration would have minimal side effects, yet it would be able to synergize with other agents even in the hypoxic areas of a tumor.

BID is thought to be cleaved by caspase-8 upon activation of receptor mediated apoptosis. Truncated BID (tBID) then translocates from the cytosol to mitochondria where it promotes release of cytochrome c and caspase-9 which, in turn, forms apoptosome and activates executive caspase-3. However, BID can also be cleaved by caspase-3 and served as a self-amplification loop (22). We suspect that BID cleavage, during VPA treatment, is mediated by caspase-3, since inhibition of caspase-8 neither prevented BID cleavage nor influenced the number of apoptotic cells.

Although HDACi have been described to trigger apoptosis through both receptor mediated (43) and intrinsic pathways, the latter was shown to be dominant in NBL cells during VPA treatment (44). We also demonstrated this in our experimental setting. We further showed that mitochondrial activation is the first event in apoptosis induction and BID cleavage and caspase-8 activation were a consequence of the progressing apoptotic cascade. Notably, there was no difference in the pathway through which apoptosis proceeded relative to normoxic or hypoxic conditions and VPA treatment.

To conclude, we showed that VPA is effective in both normoxic and hypoxic conditions and can overcome hypoxia induced resistance to CDDP-induced apoptosis. Considering all its advantages (i.e. orally applicable, low toxicity, an already approved drug), VPA alone might be beneficial in NBL treatment keeping in mind that VPA alone failed to induce significant apoptosis in some NBL cell lines. However, VPA combined with conventional chemotherapeutic drugs should be much more effective and is worthy of consideration. Additionally, VPA seems to be a very suitable compound for continued research regarding hypoxia-induced resistance. We also presented a possible role for HIF-1 as it relates to the VPA mode of action, but the direct mechanisms by which it acts are unknown and need further elucidation.

## Figures and Tables

**Table I:** Percentage of apoptotic cells measured as An^+^/PI^−^ cells.

	Control		24 h		48 h		72 h	
				
	N (%)	H (%)	N (%)	H (%)	N (%)	H (%)	N (%)	H (%)
SK-N-AS (5 mM)	1.6	1.1	5.59	6.63	13.86	14.91	13.37	19.02
SK-N-ASrCDDP (5 mM)	2.59	3.99	7.51	11.51	13.61	16.82	40.63	53.47
UKF-NB-3 (2 mM)	6.3	6.66	10.13	9.29	21.49	20.70	19.39	16.57
UKF-NB-3rCDDP (2 mM)	2.89	2.73	7.81	7.43	9.12	16.60	7.22	25.49

Concentration of VPA was 2 mM for UKF-NB-3 and UKF-NB-3 resistant to cisplatin (rCDDP) and 5 mM for SK-N-AS and SK-N-ASrCDDP. Cells were grown for 24 h under normoxic conditions before administration of VPA and before being placed into a hypoxia chamber. Similar or even lower number of apoptotic cells under hypoxic conditions in UKF-NB-3 was due to shift from An^+^/PI^−^ quadrant to An^+^/PI^+^ quadrant because of the high sensitivity of this cell line. Data from one representative experiment are shown.
